# A scoping review to map evidence regarding key domains and questions in the management of non-traumatic wrist disorders

**DOI:** 10.1177/17589983231219595

**Published:** 2023-12-12

**Authors:** Thomas Mitchell, Nick Hamilton, Ben Dean, Sarah Rodgers, Sally Fowler-Davis, Sionnadh McLean

**Affiliations:** 1 Health Research Institute, 7314 Sheffield Hallam University, Sheffield, UK; 2 Nuffield Department of Clinical Neurosciences, 6396 University of Oxford, UK; 3 The Hand Unit, 105628 Northern General Hospital, Sheffield, UK

**Keywords:** Non-traumatic wrist disorder, wrist pain, wrist injury, clinical pathways, conservative management

## Abstract

**Introduction:**

Non-traumatic wrist disorders (NTWD) are commonly encountered yet sparse resources exist to aid management. This study aimed to produce a literature map regarding diagnosis, management, pathways of care and outcome measures for NTWDs in the United Kingdom.

**Methods:**

An interdisciplinary team of clinicians and academic researchers used Joanna Briggs Institute guidelines and the PRISMA ScR checklist in this scoping review. A mixed stakeholder group of patients and healthcare professionals identified 16 questions of importance to which the literature was mapped. An *a-priori* search strategy of both published and non-published material from five electronic databases and grey literature resources identified records. Two reviewers independently screened records for inclusion using explicit eligibility criteria with oversight from a third. Data extraction through narrative synthesis, charting and summary was performed independently by two reviewers.

**Results:**

Of 185 studies meeting eligibility criteria, diagnoses of wrist pain, De Quervain’s syndrome and ulna-sided pain were encountered most frequently, with uncontrolled non-randomised trial or cohort study being the most frequently used methodology. Diagnostic methods used included subjective questioning, self-reported pain, palpation and special tests. Best practice guidelines were found from three sources for two NTWD conditions. Seventeen types of conservative management, and 20 different patient-reported outcome measures were suggested for NTWD.

**Conclusion:**

Substantial gaps in evidence exist in all parts of the patient journey for NTWD when mapped against an analytic framework (AF). Opportunities exist for future rigorous primary studies to address these gaps and the preliminary concerns about the quality of the literature regarding NTWD.

## Introduction

Wrist pain is commonly encountered with an annual consultation rate of 58 in 10,000 patients in primary care in the UK.^1^ Its incidence increases in people who engage in physically demanding occupations and for sportspeople where 10% have been found to have short-term pain, and 24% have medium-term pain.^2^ Non-modifiable associations with wrist pain include older age and female sex.^2^ In the UK’s National Health Service (NHS), a typical clinical pathway for patients with wrist disorders would see initial diagnosis and management in primary care, with referral through a musculoskeletal service for further diagnostic assessment and treatment in secondary care based on clinical need.^3,4^

The main pathological causes of non-traumatic wrist disorders (NTWD) include carpal osteoarthritis (OA), tendinopathies (including De Quervain’s and intersection syndrome), ulnar-sided wrist pathologies (itself made up of sub-groups with poor diagnostic validity),^5–11^ and ganglion. NTWD are distinct from basal thumb and hand osteoarthritis based on the clinical burden, risk factor profile, clinical relevance of synovial inflammation and established therapeutic interventions, however there is a degree of overlap between pantrapezial and wrist OA.^12,13^ Other presentations considered distinct from NTWD due to established diagnostic criteria, and condition-specific care pathways and management strategies include rheumatoid arthritis,^14^ carpal tunnel syndrome^15–17^ and complex regional pain syndrome,^18^ and were excluded from this study.

Uncertainty in various parts of the journey of care for people with wrist and hand pain was highlighted in a James Lind Alliance Priority Setting Partnership.^19,20^ Specifically, stakeholders identified a need for more information on which surgical and conservative methods enable efficient return to function, and which patient-reported outcomes are most useful in measuring the effectiveness of management. Although this review is limited to non-surgical literature these findings were a motivating factor to assessing the literature regarding NTWD in this review. In order to set research questions that are derived from stakeholder and patient involvement,^21^ this review convened a mixed stakeholder group to identify key areas for investigation.

The NHS Long Term Plan^22^ recommends patient-centred models of care through ‘shared decision making’ over historic paternalistic models which emphasise expert opinion and passive care and states this is a pressing need as rates of non-communicable diseases rise and questions around musculoskeletal management are increasingly raised.^23^ Lewis and O’Sullivan caution that trends toward an unwarranted specific musculoskeletal diagnosis in non-traumatic conditions are placing strain on health budgets and may divert resources from ‘high-value’ person-centred care, to the prioritisation of ‘low-value’ interventions targeted at uncertain diagnostic categories.^24^ When evaluating musculoskeletal care for the shoulder, low back and knee, pathoanatomical diagnosis frequently fails to explain the sufferers’ pain experience and disability in non-traumatic disorders, nor improve outcomes, leading to recommendations for the use of grouped conditions to frame management.^25–27^ This viewpoint has synergy with The Management of Wrist Pain Group (MOWP) which suggests grouping specific non-traumatic wrist diagnoses into a broader category of non-traumatic wrist pain^28^ to promote holistic rather than lesion-specific management. Through mapping the literature of all diagnoses making up NTWD, the areas of strength for particular categories can be identified.

This scoping review aims to identify the evidence for the diagnosis, management, pathways of care and outcome measures for both grouped and individual NTWD and produce a coherent and comprehensive map of key evidence gaps to direct future research.

## Methods

A protocol for the review was registered on the Open Science Framework prior to conducting any searches: https://osf.io/mxz59/.

### Study team composition

The review team of six comprised clinicians, academics, subject area specialists and a design-led expert.

### Scoping review framework

Scoping review methodology allows a systematic approach to map evidence into poorly understood areas^29^ and draws on evidence from both empirical research and grey literature sources. The current study adopted the academic standard Joanna Briggs Institute (JBI) methodology^29^ for scoping reviews and the PRISMA-ScR checklist.^30^

### Developing a rationale and identifying the research questions

**Figure 1.**
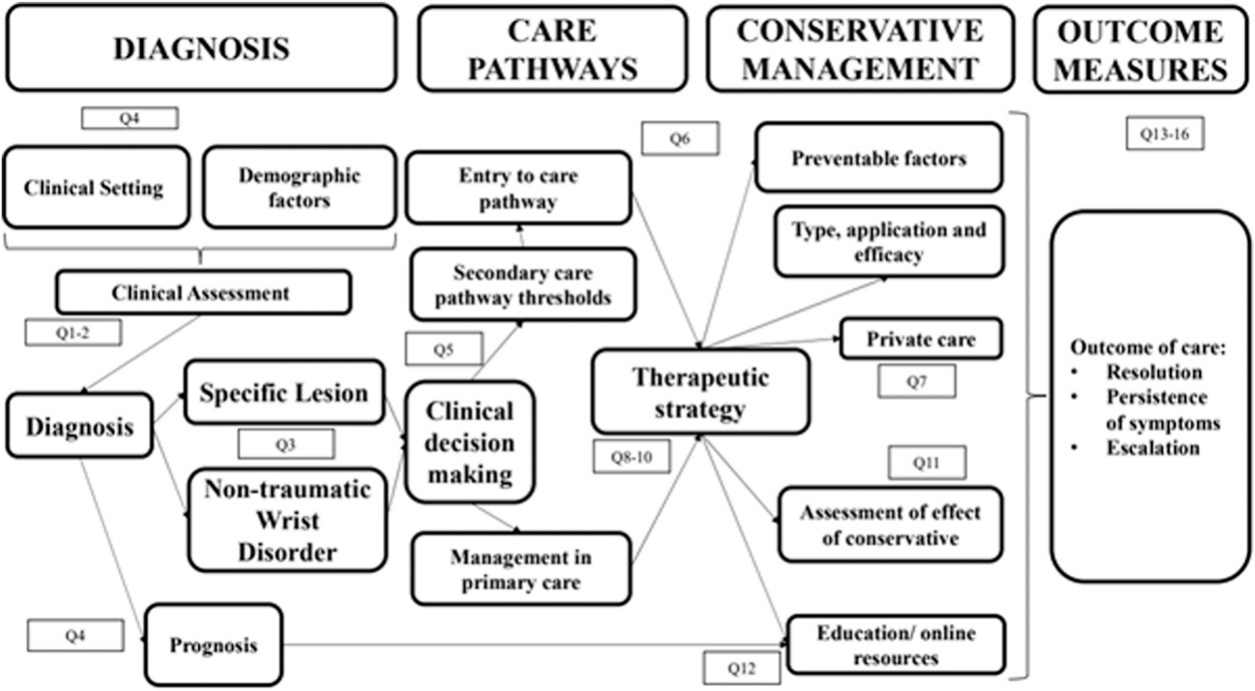
Analytic framework based on clinical journey of NTWD with key domains and related research questions.

Although stakeholder involvement is not a requirement of scoping reviews, we considered that the engagement of both people suffering with NTWD, and providers of care was important to create a robust knowledge map. Individual interviews were conducted with three people diagnosed with NTWD, one primary care clinician, one secondary care clinician and one service commissioner. A video explaining the initial aims of the review and asking for their opinion about what questions are important to investigate in the field was shown to interviewees at the beginning of the consultation. A thematic narrative synthesis of interview transcripts, using an inductive approach,^31,32^ identified 16 research questions regarded as important by stakeholders. These questions were grouped into the four domains of interest (diagnosis, conservative management, pathways of care and outcomes) (Table 1) allowing an Analytic Framework (AF) to be produced which the literature was mapped against (Figure 1).

**Table 1.** Research domains and questions identified from stakeholder interviews.

Domain	
**A**	**Diagnosis**
Q1	What elements comprise the diagnosis of non-traumatic wrist disorder and how are they staged?
Q2	What is the performance of diagnostic methods for specific structural diagnosis?
Q3	Does specific diagnosis alter management?
Q4	Do diagnoses differ based on patient demographics, duration of symptoms clinical setting or the clinician’s role and experience?
**B**	**Pathways of care**
Q5	What are the care pathways for non-traumatic wrist disorders, do they differ between settings and how are they compiled?
Q6	What are the diagnostic criteria required for entry into care pathways and what features of wrist presentations inform escalation or removal from the pathway?
Q7	How does private provision of care differ from National Health Service and how does it fit within care pathways.
**C**	**Conservative management**
Q8	What conservative management is delivered for non-traumatic wrist disorder?
Q9	Which interventions are most cost-effective and time efficient and does patient choice influence interventions selection?
Q10	Does any clinical setting show superiority?
Q11	Do pathways align with best use of interventions, and how do you know when an intervention has been effective and how does this feed into ongoing care?
Q12	Where are the best patient resources held, and are the messages consistent with best practise?
Q13	How long does it take to get better from non-traumatic wrist disorder?
**D**	**Outcome measures**
Q14	Which measures are used and what are their reliability, validity, and responsiveness for non-traumatic wrist disorder?
Q15	Is there a difference between outcomes of surgery, conservative care, and sham, and does more care equate with better outcome?
Q16	How do outcome measures inform management, diagnosis or assess effectiveness of interventions?

### Identifying relevant studies

The three-step method recommended by the JBI guidelines for scoping reviews was followed. The search strategy was intended to be broad to identify both published and unpublished (grey) literature. Initially a narrow search of Google Scholar and MEDLINE database was used to identify key literature with additional studies identified through a snowballing technique using SCOPUS to identify related literature and citations from more recent work. The University of Tasmania guide to developing search terms^33^ was applied, and we performed an analysis of the found studies to identify text words and key words relevant to the area. MeSh terms were collated via Medline to create the search strategy for Step 2 of the review (Supplementary section - 1). We applied this to MEDLINE then to PUBMED, OT Seeker, PeDRO and SPORTDiscus with minor adaptations made ad hoc to fit database requirements. Finally, a snowballing technique and citation search from key articles using SCOPUS was applied, combined with asking experts in the field for their recommended papers.

To allow non-journal and grey literature sources to be identified, we searched a variety of ‘grey literature’ databases and library catalogues via OpenGrey and Library Hub Discover (formally COPAC). Doctoral theses were identified via Ethos and ProQuest and clinical trial registers were screened via the World Health Organisation International Clinical Trials Registry Platform and the NIHR’s ‘Be Part of Research’ platform. Finally, clinical resources from BMJ Best Practice, NICE Evidence Standards Framework, the Cochrane Library and Trip were searched.

### Study selection

The titles and abstracts of studies identified in the literature search were uploaded into Covidence systematic review software (Veritas Health Innovation, Melbourne, Australia) and duplicates removed.

Explicit a priori eligibility criteria (Supplementary Section 1) were applied at Level 1 (title and abstract) and Level 2 (full text) screening. A record was included if it provided an answer to at least one of the 16 research questions (Table 1) in the analytic framework (Figure 1) and did not meet any of the exclusion criteria. The Population, Concept and Context (PCC) method proposed by JBI were used to guide the identification and inclusion of published studies of all methodologies.

#### Population

Studies of people diagnosed with wrist disorders without traumatic origin were identified as the population. Those with clear history or radiological evidence of substantial trauma were excluded (i.e., scaphoid fracture/distal radius fracture/fracture clinic patients). Those with a history of less substantial minor trauma were not excluded. People who have had surgical management for their non-traumatic wrist disorder were excluded as were athletes.

#### Concept

The concept of interest in this review included the means of assessment for non-traumatic wrist disorders, conservative/non-operative interventions commonly administered, and the pathways of care and outcome measures used in primary and secondary care.

#### Context

This review considered both primary and secondary healthcare settings as each are part of the continuum of care for non-traumatic wrist disorders in the UK. The research questions arose from the consultation exercise to allow a broad approach considering the patient journey, clinician input and wider management structures.

### Screening

Material that appeared to meet the initial screen of title and abstract was retrieved as complete reports and matched against the inclusion criteria leading to acceptance or discard by two reviewers (TM & SR), followed by a review of the full texts (TM & SR). Disagreements that arose between the reviewers were resolved through discussion, or consulting with a third reviewer (BD).

### Data extraction and synthesis

The two primary researchers (TM & SR) extracted the following data for each included article:

• Authors, year of publication, country of origin.
• Research question, aims and domains selected by study authors.
• Methods: study design.
• Participants: number of participants included, eligibility criteria, sociodemographic data (sex and age).
• Non-traumatic wrist disorder diagnostic method.
• Pathways of care.
• Management modality administered including its parameters.
• Outcome measures and indications for escalation discharge or self-management.

Literature was appraised using qualitative synthesis and related to the analytic framework and its key domains and research questions. A knowledge map assessing the extent to which the analytic framework met by the literature was formed and used to identify gaps in the literature and research priorities in future clinical research.

## Results

**Figure 2.**
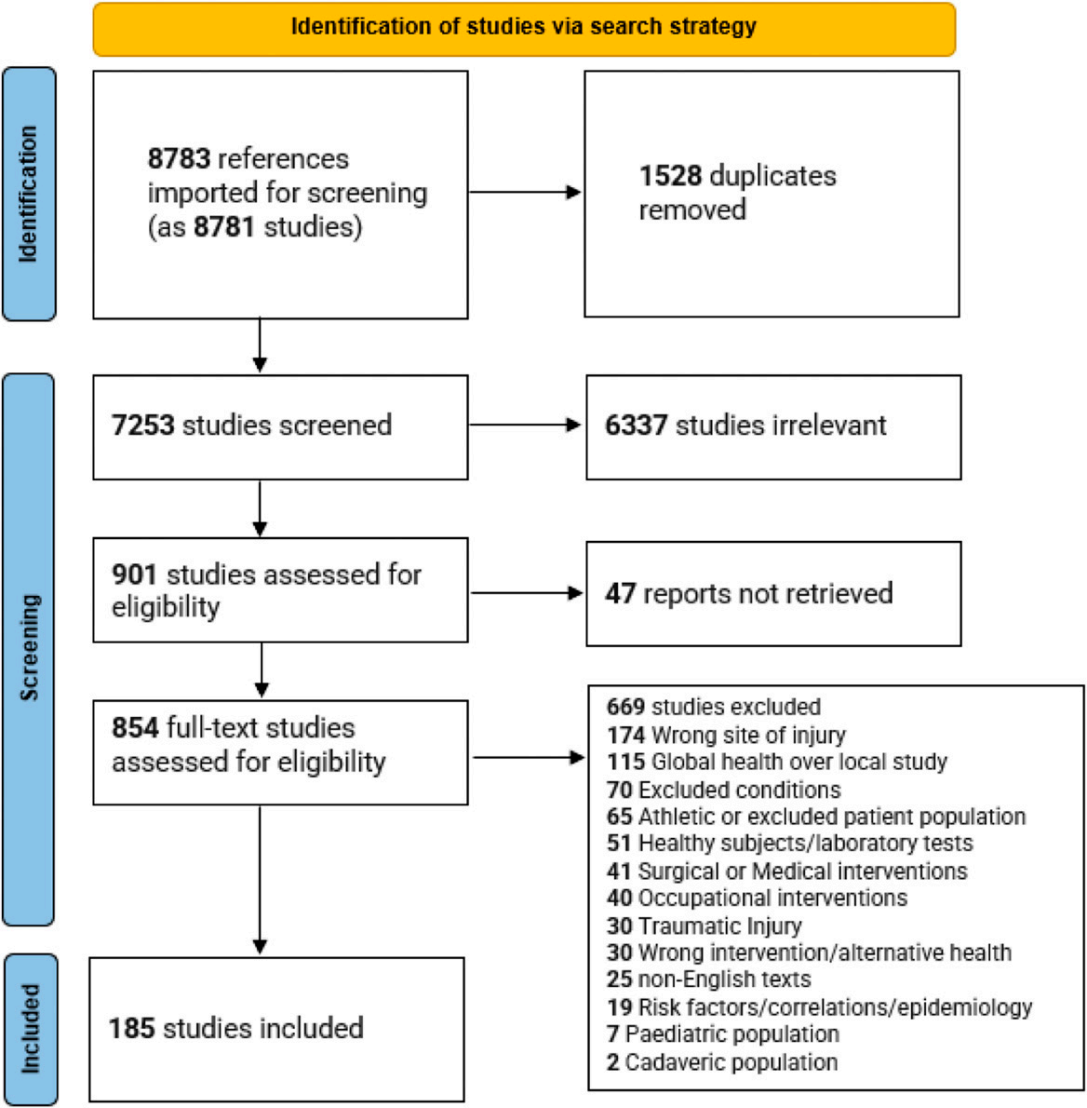
PRISMA flow diagram for NTWD scoping review.

The initial search strategy identified 8767 documents, with 16 added from a search of cited references giving a total of 8783 sources (Figure 2). 1528 duplicates were removed, and screening of titles and abstracts resulted in 901 documents selected for full-text assessment. There were 47 instances where full text was unavailable, 669 did not meet the eligibility criteria, leaving 185 suitable for inclusion (Supplementary Section 2).

The general study characteristics (Table 2) of the sample identified the most common conditions referenced were wrist pain (44 studies, 23.8%), de Quervain’s (38 studies, 20.5%) and grouped ulna-sided diagnoses were represented in 22 articles (11.9%). Where the setting was recorded, secondary care was present in 107 texts (57.8%), primary care in 11 texts (5.9%), community services in nine texts (4.9%), private practice in six texts (3.2) and mixed settings in 10 texts (5.4%). Uncontrolled nonrandomized trials (71/38.4%) were the most common study design, of which 11 (5.9%) were prospective studies, 33 (17.8%) were retrospective cross-sectional studies and 24 were case series (13.0%). Case reports accounted for 27 sources (14.6%). There were 16 systematic reviews (8.1%) of which 7 focused on de Quervain's and regarding wrist pain and ganglion respectively, seven literature reviews (3.8%) and a single scoping review (0.5%). 17 ‘clinician guides’ which uncritically describe how-to perform assessment or treatment (9.2%) were identified, and opinion pieces made up eight texts (4.3%).

**Table 2.** General study characteristics.

Study characteristics.		Studies *N* = 185	(%)
Geographic origin.			
USA	^7,8,34–71^	**40**	**21.6**
UK	^72–94^	**22**	**11.9**
Australia	^10,95–103^	**13**	**7.0**
Netherlands	^6,104–115^	**12**	**6.5**
Italy	^116–124^	**8**	**4.3**
Other		**90**	**48.6**
Main presentation of interest.			
Wrist pain	^5,10,34,38–40,45,50–52,54,55,62,63,65,67,68,71,76,80,88–90,99,102,103,106,109–111,114,125–141^	**44**	**23.8**
De Quervain’s	^36,42–44,46,47,59,72,74,77,78,85,93,97,97,105,108,119,122,131,142–161^	**38**	**20.5**
Ulna-sided	^8,9,48,58,66,87,94,113,118,162–175^	**22**	**11.9**
Carpal instabilities	^35–37,57,64,69,95,100,104,107,176–181^	**14**	**7.6**
Intersection syndrome	^49,53,60,61,121,124,182^	**7**	**3.8**
Ganglion	^44,56,73,82,115,183^	**6**	**3.2**
Nerve entrapment	^56,120,184–186^	**5**	**2.7**
Wrist OA	^75,186–188^	**4**	**2.2**
Other tendinopathy/paratendon	^92,123,189^	**3**	**1.6**
Dorsal-sided pain	^74,122,190^	**3**	**1.6**
Radial-sided pain	^70,191^	**2**	**1.1**
Red flags	^64,83,91,192–194^	**6**	**3.2**
Volar-sided pain	^7,116^	**2**	**1.1**
Care setting.			
Secondary care	^6,7,9,10,36,38,40,43–45,48–50,53,56–58,61,63,67,70–72,77,80,85–87,89,90,92,94,95,97,99,103,105–107,108–111,113,116,118,120,124,125,127,129,131,133,137,139,143–145,149–155,159,161,163–165,169–174,176–178,181–183,185,187,189,191–193,195,196,199–205^	**107**	**57.8**
Primary care	^3,13,31,41,62,65,79,118,125,135,175^	**11**	**5.9**
Community service	^34,65,102,130,138,140,165,206,207^	**8**	**4.3**
Private practice	^47,78,126,160,190,198^	**6**	**3.2**
Mixed	^45,72,77,92,97,105,149,159,197^	**10**	**5.4**
Study design			
Systematic review	^2,8,44,44,45,72,75,97,98,105,115,117,119,149,159,197^	**16**	**8.4**
DeQuervain’s	^72,97,98,105,149,159,197^	**7**	**3.8**
Ganglion	^44,115^	**2**	**0.6**
Ulna-sided	^8^	**1**	**0.3**
Wrist pain	^45,138^	**2**	**0.3**
Wrist OA	^75^	**1**	**0.3**
Neuropathy	^117^	**1**	**0.3**
Tenosynovitis (all)	^119^		
Randomised controlled trial	^55,65,103,108,131,150,151,154,161,198,199^	**11**	**5.9**
Scoping review	^37^	**1**	**0.5**
Literature review	^77,79,114,123,164,192^	**7**	**3.8**
Delphi	^156^	**1**	**0.5**
Case control study	^99,109,110,142^	**4**	**2.2**
Best practice g’line	^73,92,115^	**3**	**1.6**
Non-randomised experimental study	^67,140,144,152,153,157,178,204^	**8**	**4.3**
Uncontrolled nonrandomized trial or cohort with one or more groups	^5–7,9,10,38,40,43,49,57,58,66,76,89,101,104,107,111,127,128,132–134,137,146,167,171,172,174,181,182,195,^ ^46,48,51,70,71,74,85,95,113,121,124,125,163,173,176,177,180,183,187–191,201,202^	**71**	**38.4**
Prospective cohort study	^50,93,96,129,143,147,158,162,168,185,208^	**11**	**5.9**
Retrospective cross sectional study	^5–7,9,10,38,40,43,49,57,58,66,76,89,101,104,107,111,127,128,132–134,137,146,167,171,172,174,181,182,195^	**33**	**17.8**
Case series	^46,48,51,70,71,74,85,95,113,121,124,125,163,173,176,177,180,183,187–191,201,202^	**24**	**13.0**
Case report	^36,47,53,56,60–64,78,91,94,118,120,122,130,139,145,148,160,165,179,184,193,194,200,206^	**27**	**14.6**
Opinion pieces	^35,42,54,59,69,106,141,209^	**8**	**4.3**
Clinician guides	^39,41,52,68,80,81,83,84,86–88,116,129,169,175,196,210^	**17**	**9.2**
Qualitative research	^100,102,135^	**3**	**1.6**
Demographics of study sample subjects.			
Sex			
Mixed/not sex selected	^36,41–44,46,47,49,53,56,58–61,66,70,72,74,77–79,82,85–87,91,93,94,97,108,115,119–122,124,124,129,142–146,148–156,156–166,168,182,183,185,187,190–193,196,197,199,204^	**77**	**41.6**
Female only	^36,78,99,102,142,145,153,160,165,178,198^	**11**	**5.9**
Male only	^47,62,91,122,194^	**5**	**2.7**
Sex unclear or not reported	^5–7,9,10,34,38,40,45,50–52,54,55,63,65,67,68,71,73,76,80,81,83,84,88–90,96,98,99,101–103,106,109–111,113,114,116–118,125–137,139–141,167–175,186–188,194,195,200–206,208–211^	**84**	**45.4**
Age			
Mixed age groups	^37,38,46,48,50,57,72,75,85,86,93,95,97,97,103,109–111,113,119,124,125,127,129–131,136,137,143,144,149,153,156,157,163,171,181,188–190,202^	**40**	**21.6**
18–34	^63,76,99,102,126,134,139,142,160,165,176,177,183,194,198^	**15**	**8.1**
35–54	^7,40,47,49,51,61,62,67,71,90,122,128,133,145,150–155,158,159,161,162,187,191,195,199,201^	**29**	**15.7**
55–70	^36,65^	**2**	**1.1**
70+	^34^	**1**	**0.5**

### Studies that addressed research questions in domain A: diagnosis

Methods of diagnosis (Supplementary Section 3) were commonly stated (Q1) with subjective questioning, (36, 19.5%), self-reported pain (35, 18.9%) and heat maps (1, 0.5%) representing patient-described symptoms. The use of special tests (51, 27.9%), palpation (33, 17.8%), range of motion assessment (28, 15.1%), manual accessory motion (20, 10.8), grip (7, 3.8), weighing scale and push-off tests (2, 1.1%), and laterality assessment (1, 0.5%) represent clinician-dependent examination methods stated. The range of individual special tests referred to in the literature numbered 25. Finklestein’s was the most commonly mentioned special test, however divergence in how this was described and confusion between this and Modified Eichoff was noted.^157,158^ Six studies (3.2%) described algorithms for the staging of the methods of assessment. MRI/MRA scans (35, 18.9%), X-ray (30, 16.2%), ultrasound scan (24, 13.0) nerve conduction studies (5, 2.7%), CT scans (3, 1.6%), and arthroscopy (11, 5.9%) were the most common advanced diagnostic methods found.

The performance of various diagnostic methods (Q2) were assessed in 37 papers (20%), with six studies detailing how specificity of diagnosis informed management (3.2%, Q3), of which three were best-practice guidelines, with the remaining referring to the benefits of early diagnosis,^139^ staged management of de Quervain’s^159^ and ulnar-sided wrist pain.^169^ The impact that individual patient related factors had on diagnosis was found in 17 studies (9.2%), specifically age, clinic setting and sociodemographic factors (4, 2.2%), and three for sex (1.6%) (Q4).

### Studies that addressed research questions in domain B: pathways of care

Two care pathways from the British Medical Journal were found for ganglion and tenosynovitis of the wrist (Q5) and composed through literature reviews from area experts (Q6). Other care pathways involved a chronic wrist pain algorithm, management of ganglion and a consensus document on the treatment guidelines for De Quervain’s. The interaction between private and public health provision was not explored (Q7). All pathways identified are displayed in Supplementary Section 4.

### Studies that addressed research questions in domain C: Conservative management

Seventeen different conservative/non-operative management adjuncts to manage NTWD were referenced (Q8) in (Supplementary Section 5). The most common being injection (25, 13.5%), splinting (23, 12.4%), local exercise and manual therapy (18, 9.7%), activity modification (20, 10.8%), global exercise (9 studies, 4.9%), manual therapy sensorimotor and proprioceptive training (8 studies, (4.3%).

Cost implications of investigations were raised in five studies (2.7%), one finding routine X-ray was not cost-effective, three cautioning against the costs of investigations when they rarely change management but without scaling of costs, and one suggesting ultrasound sonography represents best value in emergency departments (Q9). Cost assessment was found for the use of injection and splints as first-line treatment for de Quervain’s, but no assessment of time efficiency nor the impact of patient choice in conservative/non operative options (Q9) was found. The effect of the clinical setting was discussed in one article which found that Primary Care was as effective in the delivery of injection as secondary care (Q10). For Q13 there were three studies which looked at the expected natural history of NTWD, one related to de Quervain’s, one regarding primary care presentations and one on the expected recovery following surgical management of ulna-sided pain.

### Studies that addressed research questions in domain D: Outcomes

The recommendation for the use of, or investigation of the validity of outcome measures of interventions (Q14) was common, with 20 different Patient Reported Outcome Measures (PROMs) found (Supplementary Section 6). The Disabilities of the Arm, Shoulder and Hand (DASH) or QuickDASH^212^ (16, 8.6%) and Patient-related Wrist and Hand Evaluation^138^ (8, 4.3%) were most frequently referenced. Other methods of assessing outcomes were self-reported pain (26, 15.1%), changes in range of motion (28, 15.1%), visual analogue scale (VAS) (16, 8.6%), patient reported numerical score (6, 3.2%), grip strength (7, 3.8%) and changes on investigation findings were used in four instances (2.2%). Six studies (3.2%) were found which addressed Q15 in creating a hierarchy superiority of management approaches. Two related to de Quervain’s and the case for injection of steroid as an effective alternative to surgery, and two related to ganglion where injecting cortisone was not indicated over conservative/non-operative management. No studies were identified which specifically looked at the how outcome measures inform management (Q16).

### Mapping onto the analytic framework

Using recommended scoping review methods, the extracted data were mapped onto the research domains and key questions from the pre-specified AF (Table 3). The extent to which the questions had been addressed was appraised to make recommendations for future research opportunities.

**Table 3.** Knowledge gaps of evidence matched to the AF with suggested research opportunities.

Domain and research question		Gaps in evidence	Clinical/research opportunities
**A**	**Diagnosis**		
Q1	What elements comprise the diagnosis of non-traumatic wrist disorders and how are they staged?	Investigate how clinicians choose and stage the elements of assessment.	The components of diagnosis are frequently described with clear commonality. The justification for the selection and staging of elements is under-investigated.
		Attempt consensus on systematic assessment process and its staging.	
Q2	What is the performance of diagnostic methods for specific structural diagnosis?	Investigation of measurement properties of tests used to diagnose non-traumatic wrist disorders.	Limited and sometimes contradictory evidence found for some elements for the use of diagnostic methods.
Q3	Does specific diagnosis alter management?	Investigation of reasoning for management and staging based on diagnostic category.	Although often claimed in opinion pieces and narrative reviews, no systematic investigation for this assertion found.
Q4	Do diagnoses differ based on patient demographics, duration of symptoms clinical setting or the clinician’s role and experience?	Further examination of demographic impact on management strategies.	Minimal sources for some subgroups, but insufficient evidence to authoritatively comment.
**B**	**Pathways of care**		
Q5	What are the care pathways for non-traumatic wrist disorders, do they differ between settings and how are they compiled?	Collection and evaluation of national and international care pathways	Two non-traumatic wrist disorders subgroups have guidelines to inform care pathways. No care pathways for other subgroups or non-traumatic wrist disorders as a group, or between care settings identified.
Q6	What are the diagnostic criteria required for entry into care pathways and what features of wrist presentations inform escalation or removal from the pathway?	Collection and evaluation of national and care pathways	Present in three guidelines for two subgroups. Insufficient evidence to comment for other subgroups or non-traumatic wrist disorders as a group.
Q7	How does private provision of care fit within care pathways, and why do private clinicians offer different things?	Investigation of care commissioner’s and clinician’s experiences in private and public settings.	Insufficient evidence to comment.
**C**	**Conservative management**		
Q8	What conservative management is delivered for non-traumatic wrist disorders?	Investigate how clinicians choose and stage the elements of treatment.	Methods of treatment are frequently described. The justification for the selection and staging of elements is under-investigated.
		Attempt consensus on systematic assessment process and its staging.	
Q9	Which interventions are most cost-effective and time efficient and does patient choice influence interventions selection?	Scaling of cost for interventions based on outcomes.	Insufficient evidence to comment.
Q10	Does any clinical setting show superiority?	Examination of clinical setting on outcome.	Insufficient evidence to comment.
Q11	Do pathways align with best use of interventions, and how do you know when an intervention has been effective and how does this feed into ongoing care?	Explore the reasoning behind decision-making and care pathways for non-traumatic wrist disorders.	Insufficient evidence to comment.
Q12	Where are the best patient resources held, and are the messages consistent with best practise?	Evaluation of web-based resources.	Insufficient evidence to comment.
Q13	How long does it take to get better from non-traumatic wrist disorders?	Warrants further investigation.	Some evidence for some subgroup, but insufficient evidence to comment for other subgroups or non-traumatic wrist disorders as a group.
**D**	**Outcome measures**		
Q14	Which measures are used and what are their reliability, validity, and responsiveness for non-traumatic wrist disorders?	Attempt consensus of clinicians on which outcome measures are recommended and the method of their utility.	No suggestion of gaps in use of objective markers. Partial evidence to comment on their validity and use.
Q15	Is there a difference between outcomes of surgery, conservative care, and sham, and does more care equate with better outcome?	Large scale multi-disciplinary collaborations.	Insufficient evidence to comment.
Q16	How do outcome measures inform management, diagnosis or assess effectiveness of interventions?	Further evaluation of the use of outcome measures.	Insufficient evidence to comment.

## Discussion

The general study characteristics revealed a substantial proportion of the included sources comprised of evidence such as clinician guides, opinion pieces and case reports (52 studies, 28.1%). There is a risk that the volume of poor-quality evidence may ‘wash out’ the evidence derived from more rigorously designed and conducted studies and suggests peer learning is prominent in this field. The predominance of secondary care settings may indicate higher concentrations of research-active clinicians in secondary care rather than revealing the extent of NTWD presenting in this setting. It is likely higher rates of presentation of NTWD occur in primary and community settings at earlier stages of the care pathway that do not progress to secondary care which may be relatively under-researched. It is notable that uncontrolled nonrandomized cohort trials (71, 38.4%) were more prevalent than RCTs or experimental studies (19, 10.3%). This is driven partly by the large number of evaluations investigating the performance of advanced diagnostic machines when compared to either consultant clinical diagnosis or arthroscopic findings. The appetite for investigating new forms of scanning equipment or to validate new clinical tests for biomechanical diagnosis is consistent, however it is interesting that some authors have questioned the reductionist premise that biomechanical lesions are the modifiable factors to target in assisting patients with NTWD.^7,9,10,28,157,162,172,202^ The other reviews (systematic, literature and scoping) represent well-conducted enquiries into the methods of conservative/non-operative management of wrist disorders and provide good information on the risk factors and epidemiology of wrist pain.

There was sufficient evidence to identify the range of assessment techniques, treatment techniques and outcome measures (Q1, Q8 & Q14), however all domains have evidence gaps related to the AF questions allowing ample opportunity for further investigation. The absence of care pathways returned from searches for subgroups and NTWD as a whole reveals a pressing requirement to understand what care is currently being delivered and how a person with NTWD navigates toward optimal management.

That a plethora of diagnosis methods, conservative/non-operative management types and means of assessing outcomes have been identified, indicates a lack of consensus in best practice for navigating the journey of care for people with NTWD. Future work should prioritise the calculation of the burden of cost and care, which NTWD represents, to give staging of research priorities.

### Potential opportunities for further studies

There is a need for further enquiry amongst clinicians and patients to identify what meaningful assessment looks like and how outcomes are best contextualised. To this end, the development of best practice guides for assessment, conservative/non-operative management options and outcome measures for NTWD would be beneficial, with recommendations on their staging. Further investigation of patient information would be useful. The means of achieving this is likely to require a mix of methodologies.

### Strengths and limitations of the review

The strengths of this review include its pre-registration on the Open Science Framework. The use of an interprofessional study team, rigorous search strategy, broad sources of literature, and engagement with a mixed stakeholder group of patients and healthcare professionals to create key questions, which were refined and expanded to form an AF based on the clinical journey of patients with NTWD in the UK, add to this strength. Limitations are in part related to the nature of scoping reviews as the broad search strategy and research question resulted in the inclusion of a large number of studies of great heterogeneity. At full-text-level review, 289 sources were excluded due to ‘wrong site of injury’ or ‘global disorders’ reflecting wrist and hand being used interchangeably. The restriction of studies selected to those of the English language was a further limitation.

## Conclusion

Significant uncertainty exists across all domains of the NTWD patient journey. There is a need for knowledge synthesis to guide musculoskeletal practitioners to administer effective, evidence-based interventions at all points along the clinical care pathway. This scoping review’s findings will help guide further research and assist us in the long-term goal of generating knowledge synthesis.

## Supplemental Material

Supplemental Material - A scoping review to map evidence regarding key domains and questions in the management of non-traumatic wrist disorders Supplemental Material for A scoping review to map evidence regarding key domains and questions in the management of non-traumatic wrist disorders by Thomas Mitchell, Nick Hamilton, Ben Dean, Sarah Rodgers, Sally Fowler-Davis and Sionnadh McLean in Hand Therapy
