# Supplementary material for: A scoping review to map evidence regarding key domains and questions in the management of non-traumatic wrist disorders
Source: Hand Ther. 2023 Dec 12;29(1):3–20. doi: 10.1177/17589983231219595 (PMC10901165; doi:10.1177/17589983231219595)
Supplement: Supplemental Material - A scoping review to map evidence regarding key domains and questions in the management of non-traumatic wrist disorders [file sj-pdf-1-hth-10.1177_17589983231219595.pdf]

Supplementary section 1: Search Terms: Search term Tasmania criteria 08.06.22.

	**Concept 1**	**Concept 2**	**Concept 3**	**Concept 4**
**Key concepts**	**Non-traumatic**	**Wrist**	**Disorder**	**Management**
**Free text terms / natural language terms**	Non-specific<br>Overuse<br>Insidious<br>Repetitive strain<br>Work related	Ulnocarpal<br>Radiocarpal<br>Triangular fibrocartilage complex<br>TFCC<br>Carpal<br>Hand	Musculoskeletal<br>Disord*<br>Pain<br>Instabilit*<br>Osteoarthritis<br>Tendinopath*<br>Dequervain*<br>Ulnar side*<br>Extensor carpi ulnaris<br>Gangl*<br>Avascular necrosis<br>AVN<br>Specific<br>Diagnosis	Conservative treatment* or conservative management or non-surgical or non-operative<br>Rehabilitation<br>Intervention* or therapy or treatment<br>Physiotherapy or physical therapy or rehabilitation<br>Hand Therapy OR Occupational therapy<br>Activit*modification<br>Exercise intervention<br>Splint*<br>Secondary stabil*or Flexor carpi ulnaris or extensor carpi radialis brevis or abductor pollucis longus or flexor carpi radialis<br>Manual therapy or mobilization or manipulation or massage or osteopathy or osteopathic or chiropractic<br>Protocols or guidelines or procedures<br>NHS or national health service or uk or united kingdom
**Controlled vocabulary terms / Subject terms** (MeSH terms, Emtree terms) *Consider: explode, major headings, subheadings*	Idiopathic/<br>Repetitive motion/<br>Repetitive stress/<br>Cumulative trauma disorders/<br>Occupational stress/	Wrist joint/<br>Wrist injuries/	Disease/<br>Arthralgia/<br>Joint laxity/<br>Ganglia/	First contact/primary care or primary health care or primary healthcare<br>Secondary care or hospital/Pathway or care pathway or clinical pathway<br>Outcome measure or outcome

Supplementary section 2: Specific diagnoses included or excluded from ‘non-traumatic wrist disorder’.

Included conditions	Excluded conditions
Ganglion	Base of thumb and hand osteoarthritis
Ulna sided wrist pain	Carpal fractures
Dequervain's Intersection and other tendinopathies	Carpal tunnel syndrome
Carpal osteoarthritis	Sclerosis
Carpal instabilities	Rheumatoid arthritis and other inflammatory arthritides
Other entrapment neuropathies	Chronic regional pain syndrome
Unknown	

Supplementary section 3: Studies that addressed Domain A of Diagnosis.

Research Domains and Related Questions (Q)		Studies N = 185	(%)
**Domain A: Diagnosis**			
**Q1: Composition of diagnosis?**			
Subjective questioning	(36,40,41,48,60,61,63,65,78,80,82,84–87,92,94,100,110,114,119,125,129,137,152,153,159,160,165,169,176,177,191,194–196,199)	**36**	**19.5**
Self-reported pain	(7,36,41,55,60,61,65,82,87,102,108,109,114,119,121,125,129,133,139,142,145,148,150–152,154,155,160,161,163,165,169,187,194,198,201)	**35**	**18.9**
Palpation	(5,36,46,47,59–61,63,87,108,114,119,121,125,129,142–146,148,152,154,155,160,161,166,169,188,193,194,201,205)	**33**	**17.8**
Range of motion	(36,40,47,64,70,78,82,83,86,87,92,114,125,126,129,135,140,143,161,164,166,169,190,191,195,205,208,212)	**28**	**15.1**
Manual accessory motion	(10,36,45,47,51,62,63,78,82,86,87,101,131,140,150,159,169,178,191,202)	**20**	**10.8**
Grip test	(76,83,99,130,154,155,164)	**7**	**3.8**
Visualisation of heatmaps	(133)	**1**	**0.5**
Push off test	(67,165)	**2**	**1.1**
Weighing scale test	(190,206)	**2**	**1.1**
Laterality	(136)	**1**	**0.5**
Special tests	(5,9,10,35,47,50,57–59,93,97,108,129,131,142–144,146,154,155,157,158,162,164,168,174,193,204,209)	**29**	**15.7**
Finkelstein's	(46,50,59,63,85,97,114,114,129,129,142,143,146,150,153–155,159,161,193,199)	**21**	**11.4**
Modified Eichoff	(50,158)	**2**	**1.1**
WHAT	(50)	**1**	**0.5**
EPL extension test	(159,199)	**2**	**1.1**
Selfie test	(209)	**1**	**0.5**
Tinel's	(120,129)	**2**	**1.1**
Kirk-Watson	(5,57,94,114,153,176,191,209)	**8**	**4.3**
Finger extension test	(191)	**1**	**0.5**
Lichtman test	(116)	**1**	**0.5**
Lunotriquetral Shuck test	(10,94,121,153)	**4**	**2.2**
Triquetroulnar critical test	(94)	**1**	**0.5**
Midcarpal instability	(10,94,121,153)	**4**	**2.2**
Radial synergy test	(50)	**1**	**0.5**
GRIT	(10,100,153,204)	**4**	**2.2**
Ballottement	(94,190,209)	**3**	**1.6**
DRUJ instability test	(121,153,195)	**3**	**1.6**
TFCC/ulnocarpal Stress test	(10,94,153,190)	**4**	**2.2**
TFCC/Ulna Fovea sign	(9,121,121,166,190)	**5**	**2.7**
ECU Synergy	(94,121,168,174)	**4**	**2.2**
Ice cream scoop test	(94)	**1**	**0.5**

Lichtman test	(116)	1	0.5
ECU dislocation	(162)	1	0.5
Ulnar carpal grind test	(121,153)	2	1.1
Piso triquetral shear test	(121)	1	0.5
Screwdriver test	(190)	1	0.5
Ulnomeniscotriquetral dorsal glide test	(209)	1	0.5
Diagnostic interventions			
X-ray	(35,40,41,46,64,68,70,80–82,88,90,92,92,95,104,107,110,114,155,165,175,176,179,181,188,189,191,194,201,211)	**30**	**16.2**
Ultrasound scan	(53,82,92,93,98,116,118,120–124,127,143,145,147,148,155,162,168,182,183,200,210)	**24**	**13.0**
Nerve conduction studies	(56,120,153,184,211)	**5**	**2.7**
CT scan	(89,193,201)	**3**	**1.6**
MRI/MRA	(5,6,8,10,35,39,49,52,56,58,64,66,71,74,89,91,121,128,132,134,145,154,155,165,167,170–173,180,185,192,202,205,208)	**35**	**18.9**
Arthroscopy	(8,10,35,58,74,132,169–171,204,211)	**11**	**5.9**
Other	(49,67,103,113,137,186,206)	**7**	**3.8**
Staging of elements	(94,121,129,140,195,207)	**6**	**3.2**
**Q2 Performance of diagnostic methods?**	(5,9,10,40,50,57,58,67,70,76,89,101,121,125,128,130,134,140,146,147,157,164,167,168,170,171,173,175,176,178,180,182,189,192,207,209,212)	**37**	**20.0**
**Q3 Does specific diagnosis alter management?**	(73,92,115,139,159,169)	**6**	**3.2**
**Q4 Patient factors affecting diagnosis?**			
Age	(40,90,90,129)	**4**	**2.2**
Clinical setting	(90,108,111,203)	**4**	**2.2**
Sociodemographic factors	(40,111,153,211)	**4**	**2.2**
Sex	(38,90,129)	**3**	**1.6**
Duration of symptoms	(100,102,129)	**3**	**1.6**
Notes	GRIT = Gripping Rotatory Impaction Test, DRUJ = Distil Radioulnar joint, TFCC = Triangular fibrocartilage complex, ECU - Extensor Carpi Uulnaris		

Supplementary section 4: Studies that addressed Domain B: Pathways of Care.

**Research Domains and Related Questions (Q)**		**Studies N = 185**	**(%)**
**Domain B: Pathways of care**			
**Q5 Existing pathways?**	(73,92,114,115,156)	**5**	**2.7**
**Q6 Pathway composition?**	(73,92,115)	**3**	**1.6**
**Q7 Private provision?**	None identified	**0**	**0**

Supplementary section 5: Studies that addressed Domain C: Conservative Management

**Research Domains and Related Questions (Q)**		**Studies N = 185**	**(%)**
**Domain C: Conservative management**			
Q8: Conservative adjuncts			
Injection	(42–44,46,60,63,72,73,86,90,92,94,97,129,142,145,149,150,152,156,156,159,183,188,200)	**25**	**13.5**
Splint	(42,60,61,63,75,86,90,92,94,95,97,103,105,139,142,144,149,150,152,156,159,161,199)	**23**	**12.4**
Local exercise	(34,35,47,51,86,90,95,97,100,106,107,126,130,131,141,159,165,177)	**18**	**9.7**
Activity modification	(47,60–63,86,90,94,95,97,105,106,130,156,159,160,164,198)	**18**	**9.7**
Manual therapy	(10,36,45,51,78,90,97,105,159,160,204)	**11**	**5.9**

Global exercise	(34,90,97,106,107,141,156,159,177)	**9**	**4.9**
Sensorimotor training	(35,95,97,100,106,141,165,198)	**8**	**4.3**
Proprioceptive training	(35,90,95,97,100,106,141,198)	**8**	**4.3**
Heat	(47,55,60,97)	**4**	**2.2**
Ice	(47,60,61,97)	**4**	**2.2**
Ultrasound	(154,199)	**2**	**1.1**
Taping	(131,164,190,198)	**4**	**2.2**
Lazer	(154,155)	**2**	**1.1**
Acupuncture	(150,156)	**2**	**1.1**
Wax bath	(97,161)	**2**	**1.1**
Watchful wait	(73)	**1**	**0.5**
TENS	(151)	**1**	**0.5**
**Q9: Cost-assessment**	(38,40,46,128)	**5**	**2.7**
**Time efficiency assessment?**	None identified	**0**	**0**
**Patient choice**	None identified	**0**	**0**
**Q10: Effect of clinical setting?**	(108)	**1**	**0.5**
**Q11: Pathways aligned with intervention best practice?**	None identified	**0**	**0**
**Q13: How long does it take to get better from NTWD?**	(102,147,195)	**3**	**1.6**

Supplementary section 6: Studies that addressed Domain D: Outcome Measures

**Research Domains and Related Questions (Q)**		**Studies N = 185**	**(%)**
**Domain D: Outcome measures**			
**Q14: Type of measure?**			
Patient Related Objective Measures (PROMS)			
QuickDash/ Dash	(67,95,128,139,149,150,152,153,160,161,165,177,183,198,199,212)	**16**	**8.6**
PRWE	(95,96,107,126,135,165,207,212)	**8**	**4.3**
Purdue Pegboard test	(34,137)	**2**	**1.1**
AUSCAN	(103,137,207)	**3**	**1.6**
Canadian Occupational Performance Measure (COPM)	(34,103,207)	**3**	**1.6**
WHYMPI	(136)	**1**	**0.5**
Modified Mayo Wrist score	(178,191)	**2**	**1.1**
Eysenck Personality questionnaire	(102)	**1**	**1.1**
General Health Questionnaire	(102)	**1**	**1.1**
Dutch Arthritis Impact Measurement Scale	(108)	**1**	**1.1**
SF 36	(198)	**1**	**1.1**
SF12	(161)	**1**	**1.1**
Eysenck Personality questionnaire	(102)	**1**	**1.1**
Euro-Qol Five Dimension Questionnaire	(177)	**1**	**1.1**
Stroop test	(137)	**1**	**1.1**
Mental rotation task	(137)	**1**	**1.1**
The Brief Illness Perception Questionnaire (B-IPQ)	(113)	**1**	**1.1**
Pain Catastrophising Scale (PCS)	(113)	**1**	**1.1**

PSFS	(36)	**1**	**1.1**
Visual Analogue Scale (VAS)	(63,95,109,126,131,137,139,144,145,149,155,157,165,190,198,207)	**16**	**8.6**
Patient reported numerical scale	(36,51,149,150,209,211)	**6**	**3.2**
Grip strength	(76,83,99,130,154,155,164)	**7**	**3.8**
Self-reported pain	(7,36,41,44,46,47,55,56,58,60,61,63,65,74,82,102,108,109,114,119,121,125,133,142,145,148–151,151,152,154,155,160,161,169,189,189,194,196,198,200,201,207,211)	**35**	**18.9**
Range of motion	(36,40,47,64,70,78,82,83,86,87,92,114,125,126,129,135,140,143,161,164,166,169,190,191,195,205,208,212)	**28**	**15.1**
Reduced symptoms on repeated physical test	(36,78,97,144,149,160)	**6**	**3.2**
Investigation change	(145,154,155,189)	**4**	**2.2**
Q15: Superiority of management?	(82,128,151,152,164,201)	**6**	**3.2**
Q 16: Measures impact on management?	None identified	**0**	**0**
Notes	WHYMPI = West Haven-Yale Multidimensional Pain Inventory, PWRE = Patient-Rated Wrist Evaluation, DASH/QuickDASH = Disabilities of the Arm, Shoulder and Hand, PSFS = Patient reported numerical score<br>SF = Short Form		
